# Transcriptome Analysis Reveals Candidate Genes for Light Regulation of Elsinochrome Biosynthesis in *Elsinoë arachidis*

**DOI:** 10.3390/microorganisms12051027

**Published:** 2024-05-19

**Authors:** Dan Liu, Jingzi Piao, Yang Li, Haiwen Guan, Jingwen Hao, Rujun Zhou

**Affiliations:** Department of Plant Pathology, College of Plant Protection, Shenyang Agricultural University, Shenyang 110866, China; liudan08266@163.com (D.L.); piaojingzi@syau.edu.cn (J.P.); liyang20210713@163.com (Y.L.); guanhaiwen0414@163.com (H.G.); hhaojingwen@163.com (J.H.)

**Keywords:** *Elsinoë arachidis*, RNA-seq, light, dark, regulation

## Abstract

Light regulation is critical in fungal growth, development, morphogenesis, secondary metabolism, and the biological clock. The fungus *Elsinoë arachidis* is known to produce the mycotoxin Elsinochrome (ESC), a key factor contributing to its pathogenicity, under light conditions. Although previous studies have predominantly focused on the light-induced production of ESC and its biosynthetic pathways, the detailed mechanisms underlying this process remain largely unexplored. This study explores the influence of light on ESC production and gene expression in *E. arachidis*. Under white light exposure for 28 days, the ESC yield was observed to reach 33.22 nmol/plug. Through transcriptome analysis, 5925 genes were identified as differentially expressed between dark and white light conditions, highlighting the significant impact of light on gene expression. Bioinformatics identified specific light-regulated genes, including eight photoreceptor genes, five global regulatory factors, and a cluster of 12 genes directly involved in the ESC biosynthesis, with expression trends confirmed by RT-qPCR. In conclusion, the study reveals the substantial alteration in gene expression associated with ESC biosynthesis under white light and identifies potential candidates for in-depth functional analysis. These findings advance understanding of ESC biosynthesis regulation and suggest new strategies for fungal pathogenicity control.

## 1. Introduction

Throughout the prolonged evolution and interaction process between plant pathogenic fungi and their hosts, various pathogenic factors have evolved to facilitate pathogen invasion and disease transmission, mainly including enzymes, fungal phytotoxins, and growth regulators. Among these, fungal phytotoxins, produced as secondary metabolites through natural biochemical processes, are characterized by their diverse chemical structures, phytotoxic effects, and modes of toxic action [1,2]. Fungal phytotoxins exert toxicity by directly damaging plant cells or by modulating plant defense mechanisms, leading to symptoms like pathogenic necrosis, programmed cell death, increased membrane permeability, intracellular energy dissipation, and others, which are crucial for disease progression and pathogenesis [3,4,5,6]. Understanding the mechanisms through which fungal phytotoxins operate is key to deciphering plant–pathogen interactions.

Plant pathogenic fungal toxins can be classified according to their structure into categories such as alkaloids, terpenes, proteins, sugars, peptides, and perylene quinones [6]. Among these, perylene quinones (PQs) mycotoxins represent a class of polyketide toxins characterized by a five-ring conjugated chromophore structure [7]. PQs can be further categorized based on the substituent positions and chemical forms of the functional groups on the side chains of their core ring structures into flavin-type, cyclosporin-type, and other types. Common examples include phyromycin, produced by *Cladosporium phlei*; elsinochrome (ESC), produced by *Elsinoë*; cercosporin, produced by *Cercospora*; hypocrellins, produced by *Shiraia bambusicola*; alternarin and alternariol, produced by *Alternaria*; and other types such as hypericin [8]. In recent years, PQs have garnered significant attention for their role in facilitating colonization and providing a competitive advantage to major plant pathogens such as *Cercospora*, *Alternaria*, and *Elsinoë*. PQ toxins are lipophilic and can target various organelles, including the endoplasmic reticulum, Golgi apparatus, chloroplasts, mitochondria, and nuclei upon entering plant cells [9]. These toxins act as photosensitizers, reacting with biomolecules and oxygen under light exposure to generate free radicals and engage in energy transfer, producing superoxide (O^2−^), hydrogen peroxide (H_2_O_2_), hydroxyl radicals (OH^−^), and highly toxic singlet oxygen (^1^O_2_), which can lead to oxidative damage and lipid peroxidation in host cells [10].

Elsinochrome (ESC) is a red or orange perylene quinone mycotoxin produced by the genus *Elsinoë*. Being a non-host-selective mycotoxin, it has garnered attention for its highly toxic reactive oxygen species (ROS) derivatives, produced under light exposure, which are lethal to plant cells [11]. Under illumination, ESC generates singlet oxygen and superoxide molecules that are universally toxic to all cell types, causing damage to cell membranes, lipid bodies, nucleic acids, and proteins [12]. Nonetheless, the regulation of ESC biosynthesis is extremely complex, influenced by a myriad of physiological, biochemical, and environmental factors, with light exposure being a requisite for its biological toxicity and the initiation of biosynthesis [13]. ESC has been identified as a crucial virulence factor in the pathogenicity of *Elsinoë*, expediting symptom development [11,14]. However, an understanding of the biosynthetic pathways and regulatory mechanisms of ESC remains nascent. Notably, *Elsinoë, fawcettii* demonstrated high toxicity to citrus and tobacco cells under light conditions, with the polyketide synthase (PKS) gene *Efpks1* being pivotal in toxin biosynthesis [12]. *E. arachidis* revealed ESC as a virulence factor whose biosynthesis is light-responsive [15]. Analysis of key genes within the *PKS* gene cluster highlighted the role of *ESCB1* in mediating ESC biosynthesis under both light and dark conditions [15]. The biosynthesis and regulatory mechanisms of ESC are currently unclear, necessitating prompt analysis to devise effective prevention and disease control strategies.

Light profoundly influences the biosynthesis and regulatory patterns of ESC. Consequently, this study leveraged RNA-seq technology to investigate the effects of light and dark conditions on the entire transcriptome of *E. arachidis*. The objectives were to (1) construct a transcriptome dataset with high-throughput sequencing technology, informed by genome sequencing, and annotate the identified genes; (2) conduct differential gene expression (DEG) analysis; (3) pinpoint candidate genes implicated in the biosynthesis of ESC and its regulation by light. These efforts aim to enhance the understanding of light-regulated mechanisms in Ascomycota fungi and provide a theoretical basis for the elucidation of the light regulation network.

## 2. Materials and Methods

### 2.1. Strain and Culture Conditions

The *E. arachidis* strain was obtained from the Department of Plant Pathology and the Key Laboratory of Plant Diseases at Shenyang Agricultural University. The strain was stored at −80 °C in a 20% glycerol solution at Shenyang Agricultural University. Dark and white light conditions were established separately, and the strains were inoculated onto PDA medium using the mycelial suspension method for constant temperature incubation at 25 °C. The dark treatments were incubated under dark conditions for 28 days, while the white light treatments received illumination at an intensity of 30 μE m^−2^s^−1^ for the same duration [11]. The growth rate and ESC content of the cultured pathogens were then assessed.

### 2.2. Extraction of ESC

The extraction and determination of toxins were performed using ultrasonic extraction and spectrophotometric methods. *E. arachidis* was cultured at 25 °C for 28 days under dark or light conditions. Forty mycelial discs were collected using a 5 mm punch, placed in 50 mL centrifuge tubes, and dried in an oven for 24 h. Acetone served as the extraction reagent, with 10 mL added to each tube. Toxins were extracted via ultrasonic vibration at 55 °C, with an extraction time of 60 min and power of 100 W. The toxin content for each treatment was calculated by comparing the absorption peak of the extract to a standard curve [11,14].

### 2.3. RNA Isolation and Sequencing

The experimental strains were inoculated on PDA medium covered with glass paper using the fungal suspension method and cultured in the dark at 25 °C for 14 days. Two treatments, dark and white, were established, with the dark treatment serving as the control. Continuous cultivation under dark conditions and exposure to white light at 30 μE m^−2^s^−1^ for 36 h was followed by the collection of mycelium. The collected mycelium was immediately frozen in liquid nitrogen and stored at −80 °C until RNA extraction [16]. Three independent samples were collected for subsequent RNA extraction and sequencing. Total RNA was extracted using the Promega RNA simple kit (Prolomag, Shanghai, China). RNA degradation and contamination were monitored using 1% agarose gel electrophoresis. For high-throughput sequencing, three biological replicates from dark and white conditions were processed. Total RNA served as the starting material for RNA-seq sample preparation. After verifying the purity of the RNA, the samples were sent to Beijing Biomarker Technology Co, Ltd. (Beijing, China) for de novo assembly transcriptome sequencing and library construction. Differential gene expression analysis was performed using the Biomarker Cloud platform (http://www.biocloud.net/) (accessed on 27 February 2024).

### 2.4. Data Analysis

In terms of quality control, the internal software Baimaike Cloud Platform BMKCloud (www.biocloud.net) (accessed on 27 February 2024) Analysis, which was developed by Beijing Baimaike Cloud Technology Co., Ltd. (Beijing, China), was employed to identify reads prone to errors and those containing adapter sequences; the associated read pairs were subsequently removed. Differential analysis of genes, based on their count values in each sample, was performed using DESeq2 R4.1.2 software [17]. This analysis facilitated the categorization of genes into upregulated or downregulated groups by comparing the relative expression levels between two sample sets. The criteria for identifying significantly differentially expressed genes were established with a threshold of fold change ≥1 and a false discovery rate (FDR) <0.01 [17]. Genes of interest were pinpointed through BLAST (https://blast.ncbi.nlm.nih.gov/Blast.cgi) (accessed on 5 March 2024) searches or searches in the local InterPreScan (https://www.ebi.ac.uk/interpro/download/interproscan/) (accessed on 5 March 2024) database [18]. Cumulative gene expression data were utilized for hierarchical clustering analysis and heat map construction, employing GeneCluster 3.0 [19].

### 2.5. RT-qPCR Analysis

The hyphae utilized for real-time PCR (RT-qPCR) analysis were identical to those used in RNA sequencing, following the previously described RNA extraction method. Subsequent treatment involved DNA decontamination using column DNase, followed by cDNA synthesis as per the Kangwei Century (Beijing, China) HiFiScript cDNA Synthesis Kit’s instructions [16]. To confirm differentially expressed genes, Primer 5.0 software was utilized to design RT-qPCR primers for candidate genes based on their nucleotide sequences. Primer details are provided in Table S1 (Synthesized by Sangong, Shanghai China). RT-qPCR was conducted using the SYBR Green Mix (Vazyme Biotech Co., Ltd., Nanjing, China) on a Bio-Rad real-time fluorescence quantitative PCR (Hercules, CA, USA) system. Each reaction had a total volume of 10 μL, comprising 0.5 μL of each primer (forward and reverse) and 1 μL of cDNA template (10× diluted), conducted over three replicates. The amplification protocol consisted of an initial 2 min at 94 °C, followed by cycles of 5 s at 94 °C, 15 s at 58 °C, and 10 s at 72 °C for a total of 45 cycles. The GAPDH gene served as an internal reference for normalizing the total cDNA amount for each sample. The relative expression levels of genes were calculated using the 2^−ΔΔCT^ method [20], with comparison to values from strains grown in the dark as the baseline (set to 1). Differences in mRNA expression levels, with reference to control values, were assessed for statistical significance using a single sample *t*-test in Graphpad Prism version 8.0.2 for Windows.

### 2.6. Blue White Irradiation

To assess the effect of blue light on the biosynthesis of ESC in *E. arachidis*, test strains were cultivated on PDA medium and subjected to continuous blue light irradiation at an intensity of 30 μE m^−2^s^−1^ for 28 days (Blue light irradiation by means of T5LED lamp tube integrated in the bracket). This experiment aimed to evaluate the growth rate and ESC content under these conditions [20].

## 3. Results

### 3.1. Growth and Toxin Production of E. arachidis

**Figure 1:**
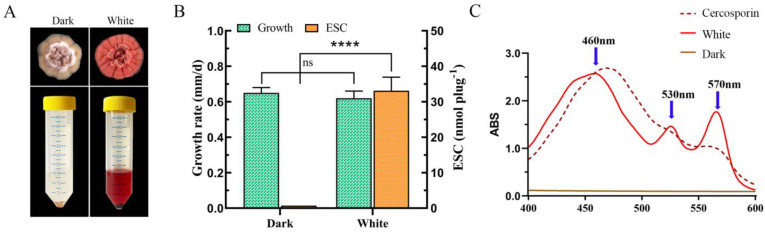
Growth and toxin production of *E. arachidis*. (**A**) Colony morphology of *E. arachidis* under white light and dark conditions, with ESC extracts below and fungal sediment at the bottom of the solution. (**B**) Measurement of mycelial growth rate and ESC content under white light and dark conditions. Growth rate is calculated as colony diameter divided by days of growth, and ESC content is derived from a regression equation. The ns and **** represent the analysis of the significance of the within-group differences. ns indicates that the difference is not significant and **** means that the *p*-value is less than 0.0001. (**C**) Absorption spectrum of ESC extract displaying a peak between 400 and 600 nm. The dashed line represents cercosporin; the solid red line shows the ESC absorption peak under white light conditions and the solid yellow line under dark conditions.

The colony morphology of *E. arachidis* exhibited significant changes between white and dark treatments; however, the growth rate showed no notable difference. In dark conditions, colonies were generally white to pale yellow in color, with the fleshy folds of the colonies protruding from the culture medium to form a volcano shape. A small amount of white flocculent aerial hyphae was observed around the base of the volcano, with a growth rate of 0.65 mm/day. Under white light conditions, the central prominence of the colony appeared fleshy and wrinkled, covered with white to light red aerial hyphae (Figure 1A). Distinct red halos were noted around the outer edge of the colony, and the growth rate was 0.60 mm/day. Moreover, significant differences in the ESC content produced by *E. arachidis* under light and dark conditions were observed (Figure 1B). The acetone extraction method facilitated the extraction of the *E. arachidis* ESC toxin under both conditions. The toxin produced under dark and white conditions was analyzed using the perylene quinone mycotoxin cercosporin as a standard. Under dark conditions, the extraction solution’s peak curve was straight with no absorption peaks in the 400–600 nm region, indicating colorless and transparent extract with no detectable ESC toxin content. Conversely, under white light conditions, the extraction solution displayed three absorption peaks at 460 nm, 530 nm, and 570 nm, revealing a clear red color with an ESC content of 30.11 nmol/plug (Figure 1C).

### 3.2. Summary of the RNA-Seq Data

Total RNA was extracted under white light and dark conditions, yielding six sequenced samples that produced 46.01 Gb of high-quality reads (Clean Data). The effective gene quality (Q30%, with a sequencing error rate < 0.1%) of *E. arachidis* was at 94.02%, and the single gene GC content stood at 54%; the specific values are shown in Table 1.

### 3.3. Analysis of Differentially Expressed Genes (DEGs)

**Figure 2:**
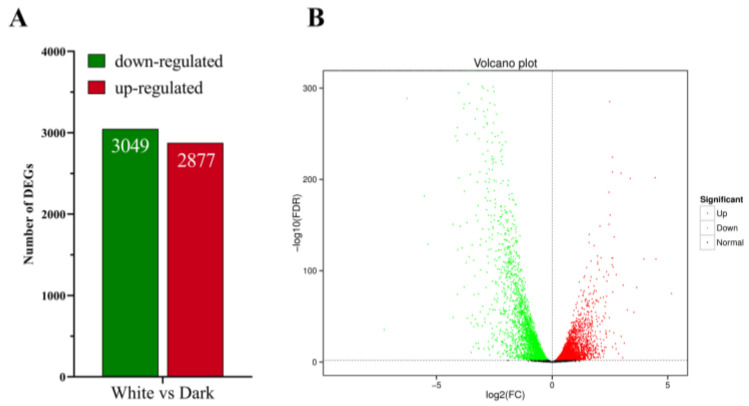
Analysis of differentially expressed genes (DEGs) in *E. arachidis*. (**A**) Differential upregulation and downregulation of genes. (**B**) Gene expression differences between white and dark conditions in *E. ararchidis*.

An analysis was conducted on the differentially expressed genes following 36 h of light treatment using *E. arachidis* treated in darkness as a control (*p* ≤ 0.05). A total of 5926 DEGs were identified, with 2877 genes upregulated and 3049 downregulated (Figure 2A). The relationship between the number of differentially expressed genes in each treatment is shown in Figure 2B. There were 1365 differentially expressed genes in the white light treatment compared to darkness, with upregulated DEGs predominantly involving keratinase (EVM0005784), aminohydrolase (EVM0006261), extracellular metalloproteinase (EVM0002471), L-amino acid oxidase (LAAO, EVM0002446), and major facilitator superfamily transporter (MFS, EVM0001084). The main downregulated DEGs under white conditions included chaperones (EVM0009088), transport proteins (EVM0007127), ribokinase (EVM0000404), and 3-dehydroshikimate dehydratase (EVM0006378). These results indicate that light influences the expression of genes related to *E. arachidis.*

### 3.4. GO Classification of Differentially Expressed Genes

**Figure 3:**
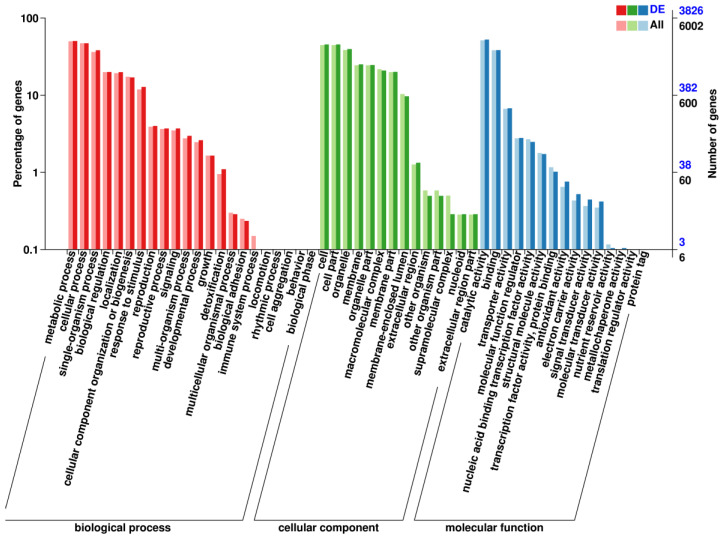
GO annotations of differentially expressed genes in *E. arachidis*. The red section represents the Biological Process (BP), the green section represents the Cellular Component (CC), and the blue part represents the Molecular Function (MF). DE is the number of upregulated genes within a secondary classification, ALL is the number of all differential genes within that classification, and the two colors are related to the value of the right y.

GO annotation of DEGs affected by white light conditions revealed a total of 4027 genes annotated in the GO database across three main functional categories: cellular component, biological process, and molecular function. In the Biological Process (BP) category, DEGs were mainly enriched in metabolic processes, cellular processes, and single biological processes. In the Cellular Component (CC) category, DEGs were enriched in cell parts, cells, and organelles. In the Molecular Function (MF) category, DEGs were primarily enriched in catalytic activity, binding, and transporter activity (Figure 3).

### 3.5. COG Analysis of Differentially Expressed Genes

**Figure 4:**
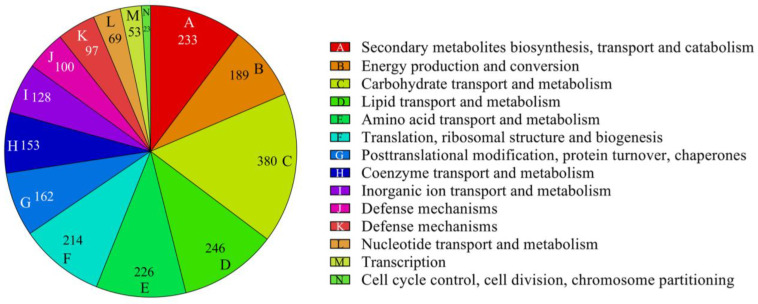
KEGG pathway analysis of transcriptomic DEGs. Each color represents a different gene category, and the number represents the number of genes within that category.

COG classification analysis was performed on DEGs, with a total of 2424 DEGs annotated into 25 COG classifications. In white light treatment, DEGs were significantly involved in processes such as carbohydrate transport and metabolism, lipid transport and metabolism, and biosynthesis of secondary metabolites and highly enriched in basic metabolic processes such as amino acid transport and metabolism, ribosomal structure and biogenesis, energy production and conversion, post-translational modifications, protein turnover, chaperones, coenzyme transport and metabolism, signal transduction mechanisms, inorganic ion transport and metabolism, and replication. Enrichment was also observed in processes related to replication, recombination, and repair (Figure 4).

### 3.6. KEGG Analysis of Differentially Expressed Genes

**Figure 5:**
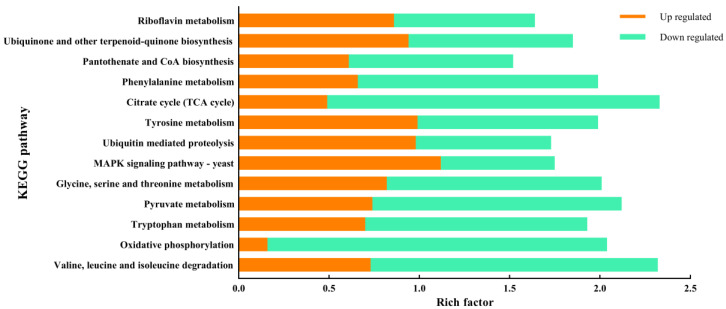
KEGG pathway analysis of transcriptomic DEGs. The y-axis indicates the function of the genes, with orange–yellow representing the number of upregulated genes and green representing the number of downregulated genes.

Based on the KEGG database, the research analyzed all the differentially expressed genes to explore the metabolic pathways of *E. arachidis* under white light regulation. A total of 1989 differentially expressed genes were mapped to 129 pathways in the KEGG pathway database, and these genes were significantly enriched in the metabolic and genetic information processing pathways. The genes affected by white light are extensively annotated in the Carbon Metabolism pathway, closely aligning with the results of the COG analysis. Extensive annotations were also found in pathways such as ribosome biosynthesis in eukaryotes, peroxisomes, degradation of valine, leucine, and isoleucine, oxidative phosphorylation, tryptophan metabolism, and pyruvate metabolism (Figure 5). In addition, a significant number of genes related to ubiquinone and other terpenoid quinone biosynthesis, the MAPK signaling pathway, and ubiquitin-mediated proteolysis were upregulated under white light compared to dark conditions.

### 3.7. Reveals Candidate Genes for White Light Regulation of Secondary Metabolism

**Figure 6:**
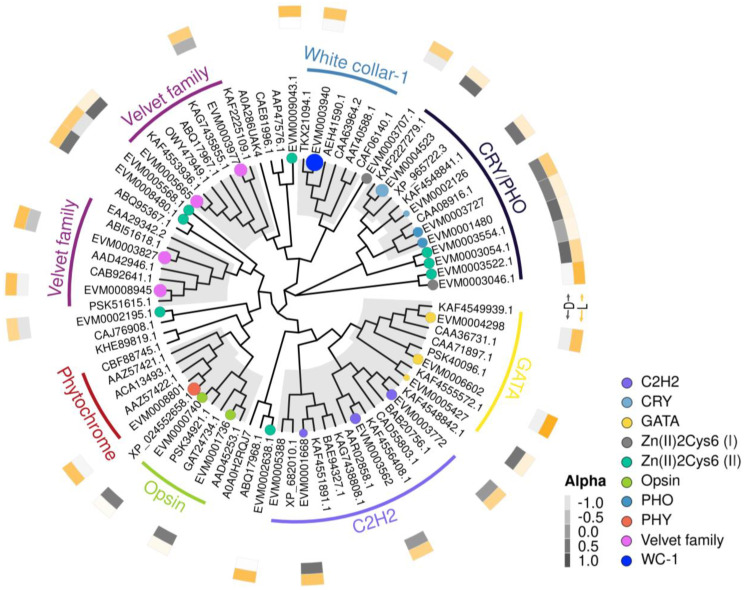
Phylogenetic analysis of genes related to white light regulation. This figure shows the phylogenetic tree of 10 genes, with the internal branch diagram of the gene, the colored dots at the end indicating genes within the transcriptome, the colored bars indicating gene classification, and the external heatmap expression of the gene under dark and light conditions.

Genes related to white light regulation may influence the level of secondary metabolism in fungi. The transcriptomic data analysis revealed overlapping differentially expressed genes. In seeking genes associated with white light regulation in *E. arachidis*, the study identified 11 putative light receptor genes among the DEGs, including 7 blue light photoreceptor genes (2 White Collar-1 genes [WC1, EVM0005181, and EVM0003940], 1 Phototropin gene [PHOT, EVM0008923], and 4 cryptochrome/photosensitive family genes [EVM0001480, EVM0004523, EVM0002126, and EVM0003727]); 1 red light receptor gene, phytochrome (PHY, EVM0008801); and 3 green light receptor genes for Opsin (EVM0002850, EVM0001736, and EVM0000740) (Figure 6).

Besides photoreceptor genes, certain transcription factors play a crucial role in signaling by directly regulating the transcriptional activation of related gene clusters, thereby affecting the biosynthesis of secondary metabolites. A total of 158 transcription factors were identified in the transcriptome, including 84 fungal-specific zinc cluster Zn2Cys6 transcription factors; 47 C2H2 type transcription factors; 5 GATA-type transcription factors; 9 bZIP-type transcription factors; 8 bHLH-type transcription factors; and 5 Velvet family transcription factors. Clustering heatmap results indicate that transcription factors are significantly upregulated under white light conditions, suggesting that a vast number of transcription factors may be activated by white light (Figure 6).

### 3.8. Genes Related to Biosynthesis of Secondary Metabolites

Light significantly affects the production of ESC toxin by *E. arachidis*. DEG analysis of transcriptome data in metabolic pathways can assist in identifying the expression patterns of genes in the perylene quinone pathway of *E. arachidis* under light treatment. Functional analysis of DEGs under white light treatment using Blast (https://blast.ncbi.nlm.nih.gov/Blast.cgi) (5 March 2024) predicted that 325 genes could be involved in the biosynthesis of secondary metabolites. According to gene functional annotation, they are primarily concentrated in nine functional groups, namely polyketide synthase, non-ribosomal peptide synthase, FAD-dependent monooxygenase, methyltransferase, FAD-dependent oxidoreductase, NADPH dehydrogenase, transporter protein, cytochrome P450, and iron reductase proteins.

**Figure 7:**
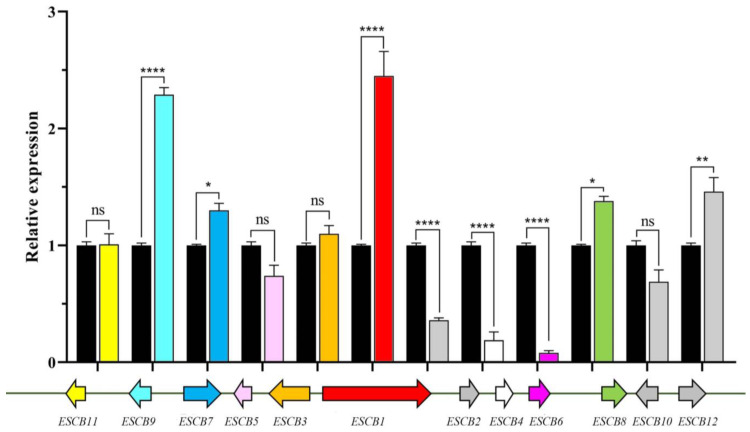
*E. arachidis* secondary metabolism gene clusters and gene expression under white light conditions. Black represents gene expression under dark conditions, and color represents gene expression under white light conditions. The upper part represents the gene expression level, the lower part represents the gene cluster and below the gene cluster is the gene name. Among them, *ESCB1* has the highest level under light conditions. The ns, *, ** and **** represent the analysis of the significance of the within-group differences. ns indicates that the difference is not significant, * means that the *p*-value ≤ 0.05, ** means that the *p*-value ≤ 0.01 and **** means that the *p*-value is less than ≤0.0001.

It is predicted that genes in the polyketide synthase (PKS) and non-ribosomal peptide synthase (NRPS) gene clusters, including eight polyketide synthases in addition to six NRPS. Blast analysis and phylogenetic tree analysis revealed that EVM0003759 is a PKS-encoding gene involved in ESC biosynthesis, EVM0004732 is a PKS-encoding gene involved in melanin biosynthesis, and EVM0005988, EVM0002563, and EVM0006869 are PKS-encoding genes involved in T-toxin biosynthesis. The gene expression patterns on the clusters showed significant up regulated expression of *ESCB9*, *ESCB1*, *ESCB8* and *ESCB12* genes under white light conditions (Figure 7).

### 3.9. RT-qPCR Analysis of Candidate Genes for Light Regulation

**Figure 8:**
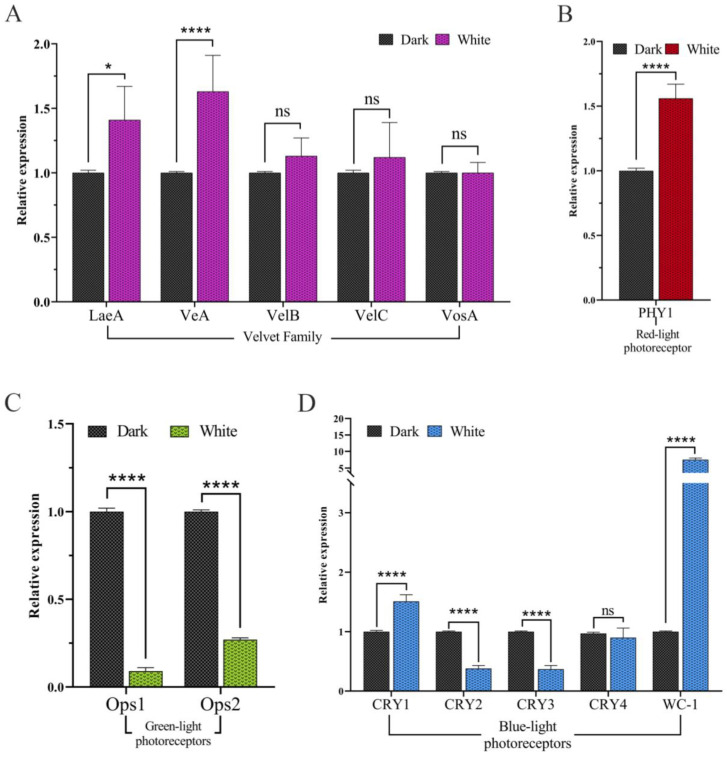
RT-qPCR analysis of candidate genes. (**A**). Expression levels of Velvet complex genes (*LaeA*, *VeA*, *VelB*, *VelC*, and *VosA*) under white light and dark conditions. (**B**). Expression level of the red-light receptor gene *PHY1* under white and dark conditions. (**C**). Expression levels of the green light receptor genes *Ops1* and *Ops2* under white and dark conditions. (**D**). Expression levels of the blue light receptor gene *WC1* and photolyase genes *CRY1*, *CRY2*, *CRY3*, and *CRY4* under white and dark conditions. The ns, * and **** represent the analysis of the significance of the within-group differences. ns indicates that the difference is not significant, * means that the *p*-value ≤ 0.05 and **** means that the *p*-value is less than ≤0.0001.

The research verified the expression patterns of selected differentially expressed genes, including photoreceptor genes and global regulatory factor-related genes, by RT-qPCR. Examination of the expression patterns of genes related to global regulatory factors has shown that compared to dark controls, VeA, VeLC, and VosA genes are significantly upregulated under white light conditions, while VeLB homologous genes are downregulated under white light conditions, consistent with the expression trend of transcriptome genes. Global regulatory factors may control ESC biosynthesis (Figure 8A).

The expression of light receptor-related genes showed that compared with the dark control, the blue light receptor genes WC-1 and CRY1 homologs and the red light receptor PHY1 homologs were significantly upregulated under light conditions, while the expression of other blue light receptors and green light receptors were downregulated under white light conditions, consistent with the expression trend of transcriptome genes (Figure 8B–D). The significant regulation of photoreceptors by white light conditions may be involved in the regulation of ESC biosynthesis.

### 3.10. The Effect of Blue Light on Toxin Production

**Figure 9:**
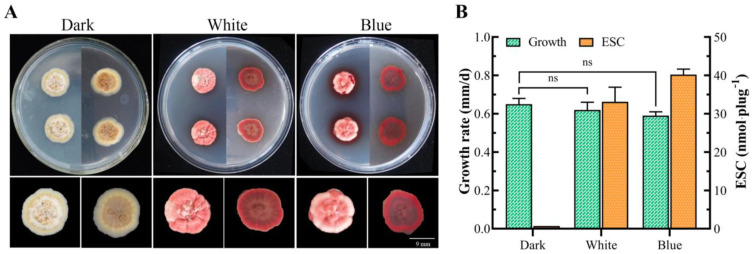
The effect of blue and white light on toxin production. (**A**). Morphological characteristics of colonies under dark, white, and blue light conditions. (**B**). Analysis of mycelial growth rate and ESC content under dark, white, and blue light conditions. The ns indicates that within-group differences were not significant when analysed.

To investigate the effect of blue light on the biosynthesis of ESC in *E. arachidis*, the strain was continuously exposed to blue light for 28 days to measure mycelial growth rate and ESC production. Under blue light conditions, significant differences in colony morphology were observed compared to darkness, with colonies appearing red with red halos; compared to white light, no significant difference was noted, but the color of aerial hyphae deepened (Figure 9A), the growth rate of hyphae slowed down, and the ESC content was 41.23 nmol plug^−1^, which is 1.24 times higher than under white light irradiation (Figure 9B). This indicates that blue light irradiation significantly positively regulates ESC biosynthesis.

## 4. Discussion

The goal of this research was to explore the genes involved in *E. arachidis* perception and response to light, applying RNA sequencing (RNA-seq) techniques. Analyzing transcriptome data under white light and dark conditions has revealed a substantial number of genes that are differentially expressed when exposed to white light. This discovery suggests that light conditions can significantly alter the transcription of genes associated with the growth, development, physiological processes, biochemistry, and secondary metabolism of *E. arachidis*. Although it is well-documented that many fungi can sense and react to light, this research marks the first instance of demonstrating significant transcriptional changes in *E. arachidis* under light-induced conditions. Moreover, it has been observed that *E. arachidis* produces a red toxin known as ESC under continuous light, with an escalation in toxin production under continuous blue light exposure. Nevertheless, the connection between this secondary metabolic reaction and the mechanisms of light signal reception and transmission has not been clearly established.

Light, being the most pivotal environmental factor in cell biology, exerts a profound influence on the growth, development, and metabolism of fungi [7,21]. This study identified that genes differentially expressed in response to light treatment were heavily concentrated in essential metabolic pathways, including the transport, biosynthesis, and degradation of carbohydrates, lipids, and amino acids, as well as the biosynthesis and degradation of secondary metabolites. These insights suggest that light conditions may significantly impact the growth, development, and secondary metabolism of *E. arachidis*. The influence of light on genes related to light-induced amino acids, carbohydrates, secondary metabolism, and stress responses has been corroborated in other fungi, like *Neurospora crassa* and *Zymoseptoria tritici*, illustrating light’s notable effect on the growth, development, and pathogenicity of fungi [9,10]. In contrast, light conditions in *Trichoderma atroviride* induce genes involved in lipid metabolism, stress response, nitrogen metabolism, localization, and biosynthesis processes yet repress genes associated with transport [11].

Marked disparities in gene expression have been observed across various fungi under the influence of light, underscoring the profound impact of photic stimuli on fungal biology [22]. Detailed transcriptome analysis of *Auricularia heimuer* under varying light intensities reveals that the augmentation of gene expression under high-intensity conditions primarily pertains to the photoreceptors responsible for light perception, the signal transduction mechanisms of the mitogen-activated protein kinase (MAPK) signaling pathway, and the melanin synthesis pathways within tyrosine metabolism [23]. This upregulation suggests these genes play pivotal roles in augmenting the production of secondary metabolites in *A. heimuer* under intense light exposure. Conversely, transcriptomic data from *Z. tritici* exposed to light and darkness indicate that while light treatments predominantly upregulated genes such as cryptochrome/photolyase CRY, rhodopsin, and components of the CWI MAPK signaling pathway, the differential gene expression observed may point to novel pathways mediating the fungal response to light [24]. In the context of this research, a total of 5929 differentially expressed genes were identified, with upregulated genes chiefly encompassing keratinase, hydrolase, oxidase, transporter proteins, and photoreceptors. This aligns with the results from GO and KEGG analyses, reinforcing the hypothesis that *E. arachidis* is responsive to light and that light exposure significantly influences the biosynthesis and transportation of secondary metabolites, though the precise molecular mechanisms warrant further exploration.

Fungi have developed sophisticated light-sensing mechanisms to adeptly navigate environmental variations, encapsulating photoreceptors, various transcription factors modulating gene expression indirectly to manage light signals, and the MAPK signaling pathways crucial for conveying light signals [25,26]. The identification of photoreceptors and associated proteins, such as flavin-bound receptors for blue light, retina receptors for green light, and linear tetrapyrrole receptors for red light in filamentous fungi, symbolizes the evolutionary adaptation to leverage these signals for activating downstream pathways [22,27]. Insights from *Fusarium* sp. species reveal the presence of at least 10 photoreceptor genes, including those encoding flavonoid proteins, photolyases, cryptochromes, rhodopsin, and photosensitive pigments, with the flavonoid protein *WcoA*, a member of the white-collar (WC) family, shown to mediate the transcriptional regulation of the majority of light-responsive genes [28]. This study identified one blue light receptor *WC-1*, four cryptochrome and photolyase enzymes (*CRY1*, *CRY2*, *CRY3*, and *CRY4)*, two green light receptors (*Ops1* and *Ops2*), and one red light receptor PHY1 in *E. arachidis*. Moreover, it was found that white light conditions notably trigger the activation of the *E. arachidis WC-1* gene alongside genes within the ESC toxin-producing cluster, hinting at a sophisticated interplay between light perception and metabolic regulation.

Research in fungal photobiology has elucidated that the regulation of *WC-1* gene expression as a photoreceptor is closely linked to the biosynthesis of various fungal secondary metabolites. However, *WC-1* also functions as a transcription factor, enabling it to perform additional roles [29]. The fungus *N. crassa* possesses a blue light receptor gene, *WC-1*, which forms heterodimers (WCC) with *WC-2*. These dimers are active under white and blue light conditions, playing a crucial role in the organism’s perception of and response to such light, notably in triggering carotenoid production [30]. Additionally, *N. crassa* features another blue light receptor, VIVID, which is minimally expressed in darkness and becomes active in light [31]. In the fungus *Alternaria alternata*, the *WC-1* homologous gene *LreA* has been identified to positively regulate the production of AOH toxin while negatively affecting the production of ATX toxin [32]. This study has unearthed the *WC-1* gene through the mining of light regulation-related genes, discovering that continuous blue light exposure boosts ESC production in *E. arachidis*. It is postulated that under light conditions, the *WC-1* gene could modulate ESC biosynthesis by functioning as a blue light receptor. The expression patterns of the *WC-1* gene suggest it may play a pivotal regulatory role in ESC metabolism, yet the specific functions of *WC-1* warrant further exploration.

The influence of light on the regulation of fungal toxin biosynthesis varies, depending on the intensity and wavelength of light, as well as the fungal species involved. In *N. crassa*, many biological processes are controlled by blue light, including carotenoid biosynthesis and circadian rhythm regulation. The absence of *WC-1* or *WC-2* genes impedes blue light perception, leading to the suppression of light-dependent carotenoid production [33,34]. In *Penicillium nordicum* and *Penicillium verrucosum*, blue light of wavelengths between 455 nm and 470 nm reduces ochratoxin A (OTA) biosynthesis by regulating the expression levels of OTA polyketide synthase [35]. In this study, the production of ESC in the peanut scab fungus increased under blue light conditions, potentially due to blue light regulating the expression levels of the ESC toxin gene cluster, thereby promoting ESC biosynthesis. The specific role and regulatory mechanism of *WC1* in this process remains to be investigated.

The biosynthesis of secondary metabolites in fungi is regulated by an extensive array of transcription factors [36]. Within this intricate regulatory network, global regulatory factors emerge as pivotal, indirectly affecting key biological processes such as toxin accumulation, virulence, and pathogenicity by coordinating the expression of genes involved in pathogenic toxin production [37]. In *Aspergillus nidulans*, light-regulated global regulatory factors have been identified, including *VeA*, *VelB*, *VelC*, and *VosA* of the Velvet family, alongside the associated *LaeA* [38]. Moreover, in *Aspergillus ochraceus*, global regulatory factors have been discovered to positively regulate the biosynthesis of the toxin OTA, significantly diminishing the pathogenicity of its mutant strains. Conversely, in *Cochliobolus heterotrophus*, these factors have been found to promote the biosynthesis of T toxins while inhibiting melanin production [39]. This article focuses on investigating five transcription factors of the Velvet family, namely *VeA*, *VelB*, *VelC*, *VosA*, and *LaeA*, through DEGs functional annotation, Blast alignment, and homologous evolution analysis. RT-qPCR analysis has revealed significant upregulation of the *LaeA* and *VeA* genes under light conditions.

Previous studies have characterized *E. arachidis* as capable of producing a photosensitive fungal toxin categorized as a perylene quinone class fungal toxin, ESC [11]. ESC is a full virulence factor in the pathogenic process of *E. arachidis*; its biosynthesis is evidently light-regulated. Further research has identified the ESC biosynthesis to be controlled by a PKS toxin-producing gene cluster encompassing 12 genes. These include the key toxin-producing gene *PKS1* (EVM0003759), O-methyltransferase encoding genes *FmOm1* (EVM0001135) and *Omf1* (EVM0007299), the transporter gene *MFS1* (EVM0006582), cytochrome P450(EVM0002495), and the pathway-specific transcriptome factor *PsTF1* (EVM0002638). This study has demonstrated that under continuous white light irradiation, the peanut scab pathogen produces ESC, and the expression of toxin-producing gene clusters is significantly upregulated under light conditions, further affirming the role of light in regulating the pathogen’s secondary metabolism.

## 5. Conclusions

This research provides a new perspective on the dynamic transcription of each gene under white light conditions in *E. arachidis*. There are significant differences in colony morphology between dark and white light conditions in *E. arachidis*, along with a substantial discrepancy in ESC production. Subsequent transcriptomic analysis identified 5926 differentially expressed genes (DEGs). GO, COG, and KEGG analyses identified 169 genes related to light regulation, including 11 putative photoreceptors. Through Blast alignment, phylogenetic tree, and domain analysis, five blue light receptor genes (with EVM0003940 as a *WC1* homolog), two green light receptor genes, one red light receptor gene, five global regulatory factors, and 12 secondary metabolism-related genes were identified. RT-qPCR results under white light conditions showed that 12 secondary metabolism-related genes were regulated by light, with the expression level of the blue light receptor *WC1* gene increasing up to 7.48-fold. Blue light irradiation experiments further confirmed that the production of ESC in *E. arachidis* under blue light conditions exceeded that under white light, possibly due to the important role played by the *WC1* gene. This study reveals light-regulated genes in *E. arachidis*, and future functional analysis of these genes may provide novel insights into the light-regulation mechanisms that are significant for the pathogenicity of this fungus.

## Figures and Tables

**Table 1:** Statistics of transcriptome sequencing.

Sample	Raw Reads	Clean Reads	Mapped Ratio (Base)/%	Q30/%	GC Content/%
D01	45,669,778	22,834,889	95.97%	94.41%	54.79%
D02	46,787,346	23,393,673	95.47%	94.02%	54.67%
D03	41,349,942	20,674,971	95.56%	94.19%	54.85%
W01	68,671,660	34,335,830	95.71%	94.19%	54.84%
W02	52,122,102	26,061,051	95.80%	94.40%	54.88%
W03	54,285,172	27,142,586	95.85%	94.31%	54.90%

## Data Availability

Data are contained within the article.

## Supplementary Materials

The following supporting information can be downloaded at: https://www.mdpi.com/article/10.3390/microorganisms12051027/s1, Figure S1: Cluster analysis of differentially expressed genes; Table S1: The information of RT-qPCR primers.
