# Supplementary material for: Transcriptome Analysis Reveals Candidate Genes for Light Regulation of Elsinochrome Biosynthesis in Elsinoë arachidis
Source: Microorganisms. 2024 May 19;12(5):1027. doi: 10.3390/microorganisms12051027 (PMC11124282; doi:10.3390/microorganisms12051027)
Supplement: Supplementary file 1 [file microorganisms-12-01027-s001.zip › Supplementary materials.pdf]

Table S1 The information of RT-qPCR primers

Primers	Primer sequence （5′-3′）	Primers	Primer sequence （5′-3′）
GAPDH-F	TCCACCCACGGTCAGTTC	GAPDH-R	TCTTTCCACGGGATGTTG
WC1F	TATCGCCATCGGCACTCA	WC1R	CGGATTCGGAAGACTACA
CRY1F	GGCGAGTCAAGACAGATT	CRY1R	GAGGAGGGAGCGAAGATG
CRY2F	AAGGACGATACCGAGAAAGC	CRY2R	CCAGTGTCGCCTCATACCA
CRY3F	AGGCGGGCATAGAAGTCA	CRY3R	CGGGCTCTGCTGTAGGTT
CRY4F	GCTGCCAAGCCTACATCG	CRY4R	TCCTTCGCCAACTTCCAC
PHY1F	GAGGGACAAGTAGTCGTCAAGCA	PHY1R	GATACCACTGAGCGATATGG
Ops1F	CTTCTCGGTCGTCTCCTTC	Ops1R	GGTGGTAACGCTCCAGTCA
Ops2F	GGGATACTTTGCCTTTGGA	Ops2R	GCCTCAGAGTTGGCGTGG
VeAF	CGATGAGAAGGCTTTGGG	VeAR	CCTTTCCGTTCTTGTCGTG
VelBF	GACATCGGCACTCACGCTTCT	VelBR	TCGTCCTTTCCGCCTCTGG
VelCF	TGCCCACGCTTTCCCATCT	VelCF	ACCCGCCTGTCTCGTTCTCC
LaeAF	GGATGTGGGACGGGTTTC	LaeAR	TTGACGGATGTCGCTGTG
VosAF	CTCGCACCACAGCAACAC	VosAR	TTCATAGGCGGGCACCAG
ESCB1	ATCCGAGGTCATTGGTGATG	ESCB1	GAGGTTGACATCTGGCATTTG
ESCB2	TTGCCAAGTTTGCCATTA	ESCB2	ACACTGCGTGAGCGACA
ESCB3	CTCGTATGCCTAAGATGG	ESCB3	AGTTCGGTGTATGAGATG
ESCB4	GCCATCGGCTTCTCCAA	ESCB4	CCGTGTTGCCTGCGTTC
ESCB5	CCTGACTTGGAATCTTGG	ESCB5	GTAGCCGTATGACTCAATAG
ESCB6	GCACAAGGTTACAGCCAAGC	ESCB6	GAAGTCGCCAAACAGAGC
ESCB7	CTACATCAATCTTCCAATCGGTC	ESCB7	GCAGGGCAAGTAGGATACAGAC
ESCB8	TACCGCTGATGATGAGTT	ESCB8	CGTCCTACACAAATACTGATTA
ESCB9	TACGATACGGCACCTCAC	ESCB9	CGATGACGAAGGAGAAGGA
ESCB10	CAGCGAGAACTCCACCG	ESCB10	ATGAGCCGAAACCGACA
ESCB11	GTCAGCCCACGAAGAAGCA	ESCB11	CAGCGAGGTAGCAAGCAAGT
ESCB12	TGATTCTGGTAACGATGATGT	ESCB12	TTGGTGGAAGATGCTGTC

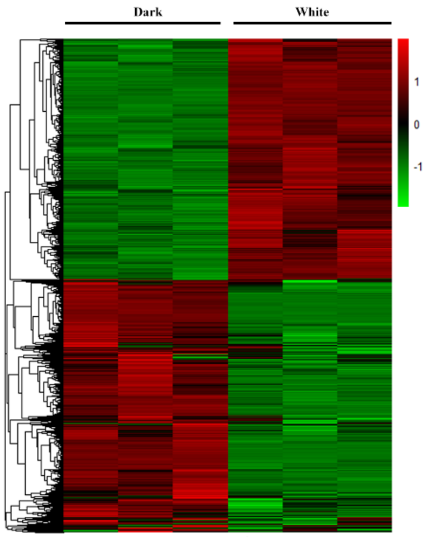

Figure S1 Cluster analysis of differentially expressed genes.
